# A Practical Perspective on the Use of Botanicals During the COVID-19 Pandemic: From Proven to Potential Interactions

**DOI:** 10.1089/jmf.2021.0062

**Published:** 2022-01-13

**Authors:** Alexander Bertuccioli, Marco Cardinali, Francesco Di Pierro, Simone Magi, Giordano Zonzini

**Affiliations:** ^1^ Department of Biomolecular Sciences, University of Urbino Carlo Bo, Urbino, Italy.; ^2^ Department of Internal Medicine, Infermi Hospital, AUSL Romagna, Rimini, Italy.; ^3^ Digestive Endoscopy Unit and Gastroenterology, Fondazione Poliambulanza, Brescia, Italy.; ^4^ Scientific & Research Department, Velleja Research, Milano, Italy.; ^5^ Italian Association of Fitness e Medicine (AIFeM), Ravenna, Italy.

**Keywords:** *botanicals*, *COVID-19*, *CYP*, *cytochrome*, *nutraceuticals*, *SARS-CoV-2*

## Abstract

In this review, we examined the top 10 nutraceutical products sold in Italian pharmacies and parapharmacies as well as hypermarkets and supermarkets; in the first, three product categories saw the greatest increase in sales (vitamins and minerals, immunostimulants, and sleep products) for the 12-month period between October 2019 and October 2020 (including first pandemic wave of SARS-CoV-2). We are investigating their respective formulas and isolating the botanicals that are used to make them. Many of these products have undergone preclinical and clinical studies. We performed a systematic literature search in the MEDLINE database using PubMed and Google Scholar from November 15, 2020 to December 15, 2020 (including studies carried out between 1980 and 2020). The search terms that were used included the complete name of the medicinal plant in English or Latin and the terms “cytochrome” or “drug interactions,” crossing, respectively, the Latin name and English common names with “cytochrome” and “drug interactions.” The search included *in vitro* and *in vivo* studies describing the effects of interaction between the plant (extract or botanical medicine) and human cytochromes. Despite their great complexity, there is decidedly limited clinical data on botanical medicine. In fact, of the 28 botanicals that were examined, only 2 (*Citrus paradisi* and *Rhodiola rosea*) show *in vivo* pharmacological interactions in human subjects. On the contrary, for the other botanicals, there is only weak evidence of dubious clinical significance or potential interactions shown in animal models or *in vitro* without clinical confirmation. This study provides a rational assessment of the most widely used products, including those used in self-medication, to simplify patient management during the COVID-19 health emergency.

## INTRODUCTION

The SARS-CoV-2 pandemic has been recognized as a potential etiological agent in mental health disorders because of its direct and indirect effects on psychological and social health. Containment measures, including quarantine and social isolation, are among the main factors that can negatively impact mental health and may play a role in acute stress disorders, anxiety, irritability, poor concentration, and indecisiveness, deteriorating work performance, post-traumatic stress disorders, high psychological distress, depressive symptoms, and insomnia. A recent survey carried out in Italy between April 19, 2020 and May 3, 2020 showed a rising incidence of mental health issues, with 24.7% of the interviewed subjects suffering from depression, 23.2% reporting symptoms of anxiety, and 42.2% suffering from sleep disorders, with 17.4% reporting moderate/severe insomnia.^1^ This also had repercussions for the nutraceuticals industry. Indeed, an analysis carried out by the National Association of Health Products Manufacturers and Distributors (Federsalus) in December 2020 showed a 38% increase in the sales volume of vitamins and minerals as well as immunostimulants, and a 28.8% increase in the sales volumes of sleep products.^2,3^ The products that were assessed are freely sold at pharmaceutical, parapharmaceutical, and supermarket and hypermarket sales points (which require the presence of personnel able to provide advice on the purchase and use of the products). These products may contain vitamins, minerals, and botanicals.

Botanicals and their derivatives are being used more and more in the Western world, as their standalone use or in synergy with prescription drugs is becoming widely accepted in the management of common illnesses. According to the latest WHO global report on traditional and complementary medicine, at least 39% of the German and British population use or have used botanicals.^4^ While many of these products have undergone preclinical and clinical studies, due to different national drug regulatory rules, it is not mandatory to study the pharmacokinetics of every botanical product. Hence, not much is known about the pharmacokinetic properties of botanicals or whether their assumption could impair the effects of other ingested drugs or *vice versa*. Indeed, the concomitant use of botanicals and prescription drugs that share the same metabolic pathway could theoretically pose a serious risk, leading to the inefficacy of many lifesaving drugs, or, even worse, leading to supratherapeutic drug exposure that could even cause fatal levels of toxicity.

Among drug metabolism and excretion components, one common pathway for many compounds is the P450 cytochrome system, a hepatic microsomal enzyme complex involved in the phase 1 reaction of the metabolic pathway. The P450 cytochrome system oxidizes not only many compounds, including drugs and botanical medicines, but also many other xenobiotics, allowing their clearance.^5^ The P450 cytochrome system is composed of many different isoforms, each one responsible for the metabolism of a specific class of molecules. CYP3A4, CYP2D6, CYP2C9, CYP1A2, and CYP2E1 are the P450 cytochrome isoforms that are most involved in drug metabolism. They are not present in equal proportions, and their representation, which is influenced by many parameters, including age, sex, and health status, is different in every individual. These isoforms can also be induced or inhibited by many exogenous molecules, including prescription drugs and botanicals. Hence, the clearance of the same amount of a given drug could vary greatly from individual to individual.^6^

Several studies^7–13^ have investigated this issue. In our investigation of reviews, systematic reviews, and metanalyses that examine botanicals and their possible interactions, several distinct approaches emerged: based on the functional classification of the botanicals^14^ or investigating several botanicals or whole herbal extracts from laboratory, animal, human subjects for their impact on cytochrome P450 isoenzymes or assessing specific drug–herb interactions within or not a clinical trial, or in case reports and case series,^10,15–18^ focusing in some works on sources, causes, and clinical relevancy of pharmacokinetic herb–drug interactions,^19–22^ or examine the top botanicals sold in the United States.^22^

The present review is the first of its kind to focus specifically on botanicals that are commercially available in Italy in a specific public health context, namely the pandemic, an already complex situation for clinicians, in which any further confounding elements could pose significant challenge.

### The real clinical question

Interactions between botanical products and conventional drugs are frequently described in the medical literature. Moreover, while results from *in vitro* studies carried out using hepatic tissue containing cytochromes may raise concerns, they do not carry the same weight as clinical trials on human subjects because of the complexity of human metabolic pathways. Furthermore, many of the available data may prove to be inconclusive. For example, Piperine, one of the principal alkaloids of *Piper nigrum*, has been shown *in vitro* to be an inhibitor of CYP3A4 and a less potent inhibitor of CYP1A2 and CYP2D6.^11,12,23^ Old studies conducted on human volunteers seem to confirm these findings, showing an increase in the levels of rifampicin and phenytoin in the bloodstream with administration of 20 mg/day of Piperine, but specific involvement of P450 cytochrome was not specified.^24^ Shedding light on this issue is of considerable importance because numerous botanical derivatives could be added to the physician's toolbox expanding the therapeutic options and allowing them to save conventional pharmacological tools for severe cases. It is therefore essential to have rationally organized and quickly readable data to be able to use these additional tools in complete safety.

## MATERIALS AND METHODS

Based on the analysis carried out by the National Association of Health Products Manufacturers and Distributors (Federsalus),^2^ we consulted the IQVIA data for the 12-month period between October 2019 and October 2020. The data analyzed come from the panels of pharmacies (8000 representative pharmacies in Italy) and parapharmacies (400 representative parapharmacies in Italy) that IQVIA manages, collects, produces, and makes available to its customers, and from the panels of super and hypermarkets managed by Nielsen, which sell botanicals with dedicated corners. This allowed us to identify the top 10 products sold, respectively, in the pharmaceutical, parapharmaceutical, and super and hypermarket channels (which require the presence of personnel able to provide advice on the purchase and use).

In the pharmaceutical channel, the top 10 products represent, respectively, 32% of the value and 28% of the volume of sedative-sleeping pills, 38% of the value and 34% of the volume of polyvitamins/minerals, and 33% of the value and 24% of the volume of immunostimulants. In the parapharmaceutical channel, the top 10 products represent, respectively, 22% of the value and 20% of the volume of sedative-sleeping pills, 29% of the value and 25% of the volume of polyvitamins/minerals, and 36% of the value and 27% of the volume of immunostimulants. Finally, in the super and hypermarket channel, the top 10 products account, respectively, for 46% of the value and 54% of the volume of sedative-sleeping pills, 46% of the value and 30% of the volume of polyvitamins/minerals, and 81% of the value and 78% of the volume of immunostimulants.

Once the products were identified, we examined their respective formulas, excluding overlapping product formulas (*e.g.,* different formats of the same product or the same product present in multiple distribution channels) and isolating the botanicals used (Supplementary Table S1).^3^

We performed a systematic literature search in the MEDLINE database via PubMed and in the Google Scholar portal from November 15, 2020 to December 15, 2020. The search terms that were used included the complete name of the medicinal plant in English or Latin and the terms “cytochrome” or “drug interactions,” crossing, respectively, the Latin name and English common names with cytochrome and drug interaction. The search included *in vitro* and *in vivo* studies, which described the effects of the interaction between the plant (extract or botanical medicine) and human cytochromes or equivalent study models.

The search encompassed studies carried out between 1980 and 2020, but focused on investigations involving human clinical trials and recent reviews. All the articles that did not include an evaluation of the *in vivo* effects on humans or animal models or *in vitro* effects on equivalent human or animal cellular study models were excluded. Out of a total of 246 articles that were found, 104 met the requirements described above. Case reports or articles which lacked data but provided information relevant to the topic in sections dedicated to the individual botanicals were also taken into consideration.

All the works that were retrieved were read, and described interactions were reported in Supplementary Table S2. The table includes the name of the medicinal plant, the cytochrome involved in the interaction, and the effect of the interaction (inhibition, induction, or generic interaction), followed by a list of commonly used drugs which could be affected by concomitant administration.

In the Results section, only references of significant importance for the purposes of the discussion were included. A paragraph for each medicinal plant highlights the main cytochrome interactions and their clinical importance that were found.

## RESULTS

### Boswellia serrata

*Boswellia serrata*, also known as Indian olibanum, is a species of plant of the Burseraceae family, native of India and the Punjab region. The dried gum-resin is the best known botanical derived from the tree; the resin and its extract contain a large amount of boswellic acid and its derivatives, which have been used traditionally as analgesics to treat joint pain, arthritis, and other forms of rheumatism.^25^ A single *in vitro* study showed how boswellic acid and its derivatives can inhibit various P450 cytochromes, namely CYP1A2, CYP2C9, CYP2C19, and CYP2D6.^26^ However, since there are no clinical studies in the literature, the significance of these interactions is questionable.

### Camellia sinensis

*Camellia sinensis* (or green tea) is a species of shrub in the flowering plant family Theaceae, and its leaves are commonly used to produce tea. Indeed, it may be one of the most widely used plants. The main substances released by its oral ingestion are teine and epigallocatechin gallate, substances with renowned stimulating and antioxidant properties.^27^ CYP3A4, CYP2D6, and CYP2C9 interactions have been described in preclinical studies using *in vitro* and animal models, pointing to possible interactions with commonly used drugs such as warfarin, midazolam, irinotecan, and tamoxifen. However, clinical studies on healthy volunteers found no interaction with CYP2C9 and CYP2D6 metabolized drugs, showing only a minor decrease in CYP3A4 activity in one study and no decrease in its activity in another investigation.^28,29^ Camellia sinensis leaf extract, was found to have CYP1A, CYP2B, and CYP3A induction capacity in animal models, while several other potential interactions have been reported *in vitro*, but the clinical significance of such reports remains unclear.^23^

### Carica papaya

*Carica papaya* (papaya, papaw, or pawpaw), a tropical fruit plant belonging to the Caricaceae family, produces spherical or cylindrical berries, 15–45 cm long, commonly eaten as a whole fruit, or used to make a juice. It is used as a botanical in its whole fruit form, or in the form of papain, a proteolytic enzyme derived from the dried fruit latex, which is used as a digestive and edema-reducing agent.^27^ Papaw juice has shown modest CYP3A4 inhibition properties *in vitro*,^30^ while whole fruit consumption could inhibit CYP2E1, as shown in an *in vivo* animal study.^31^ Although a few case reports hypothesize important interactions with drugs such as warfarin,^32^ no data derived from human clinical trials are available.

### Chamaemelum nobile

*Chamaemelum nobile* (Roman chamomile or English chamomile), a low growing perennial plant of the Asteraceae family, has been traditionally used as an infusion or as an extract for its renowned relaxing properties and as a sleep adjuvant.^25^ Our search did not find case reports or relevant P450 cytochrome interaction studies, neither *in vivo* nor *in vitro*. In fact, since *C. nobile* is widely used, drug interactions seem unlikely.^33,34^

### Citrus aurantium

*Citrus aurantium* (bitter orange or Seville orange), indigenous to tropical Asia, is a citrus tree whose fruit is commonly used as a food. The bitter orange is also used as a botanical for its neurostimulating properties, attributable to one of its constituents, the alkaloid synephrine, which has adrenergic effects.^27^ The whole fruit also contains flavonoids and furanocoumarins, which may be responsible for the CYP3A4 inhibition observed in a small clinical trial, which found a rise in cyclosporine blood levels in healthy volunteers.^35^ Moreover, a significant CYP3A4 and CYP1A2 induction was also observed in an *in vivo* animal study, showing a possible mixed effect on cytochromes.^36^ Therefore, since large human clinical trials are still not available, caution should be exercised with the concomitant administration of *C. aurantium* derivatives and CYP3A4 metabolized drugs.

### Citrus paradisi

*Citrus paradisi*, or grapefruit, is a subtropical citrus tree known for its fruit.^37^ It is traditionally used as a botanical in various forms: whole fruit, juice, extract, essential oil, or dried seed extract. All these derivatives are known for their antioxidant and antimicrobic properties.^38^ As this fruit is rich in furanocoumarins, such as bergamottin and bergaptene, it has a renowned capability to irreversibly inhibit intestinal and hepatic CYP3A4 with consequent significant interactions. In fact, it is recommended not to drink grapefruit juice with warfarin, benzodiazepines such as alprazolam, statins such as atorvastatin, immunosuppressives such as cyclosporine, antiplatelet agents such as ticagrelor, and antivirals such as indinavir because these drugs could raise the blood levels to dangerous levels, even resulting in fatal toxicity events.^11,12,39–45^ The dried seed extract was also reported to have a potent CYP3A4 and CYP2C9 inhibitory activity in a study carried out after the publication of a case report, which displayed a rise in warfarin blood levels in a patient who was also consuming grapefruit seed extract.^46^

### Crataegus oxyacantha and Crataegus monogyna

*Crataegus* (or Hawthorn) is a genus of shrubs and trees in the Rosaceae family. *Crataegus monogyna*, the most common species of the genus, is a flowering plant whose leaf and berry extracts are used for their antioxidant, cardioprotective, and cardiotonic properties. *Crataegus oxyacantha* is the name, still used in some products but no longer recognized by the botanical community, of a species of Northern European hawthorn, which was subsequently identified as a variant of *C. monogyna* and *Crataegus laevigata*.^25^ While a few case reports hypothesized the possible interaction of hawthorn with commonly used drugs such as digoxin, a clinical trial rejected this hypothesis^47^ and our search did not retrieve reported interactions with the P450 cytochrome system.

### Echinacea angustifolia

*Echinacea angustifolia* (narrow-leaved purple coneflower or blacksamson echinacea), of the Asteraceae family, is a North American plant whose root extract, rich in flavonoids, has been traditionally used as a botanical for its antioxidant properties.^25^ The presence of flavonoids could also be responsible for the plant's reported *in vitro* CYP3A4 inhibition.^48,49^ However, there is a lack of reliable drug interaction reports or clinical trials in the literature regarding this inhibition.

### Echinacea purpurea

*Echinacea purpurea* is an herbaceous flowering plant belonging to the Asteraceae family. Growing in Eastern and Central North America, it is traditionally used for the prevention and treatment of acute upper respiratory tract infections, thanks to its immunostimulant properties.^25^ The *E. purpurea root* extract shows repressive capacity on CYP2D6, CYP3A4, CYP2C19, and CYP2C9 *in vitro* and on CYP1A2 and CYP3A *in vivo* in humans, while *E. purpurea whole plant extract* shows CYP3A1 and CYP3A2 inhibition and *in vivo* CYP1A1 and CYP2D1 induction in animal models. Given the widespread consumption of this botanical, the real clinical significance of these interactions remains questionable.^50,51^

### Eleutherococcus senticosus

*Eleutherococcus senticosus* (devil's bush or Siberian ginseng), a small woody shrub native of north-eastern Asia, has traditionally been used as a botanical, as its root extract is used to make a renowned tonic and antistress agents.^25^ While its root extract has shown CYP2C9 induction capability *in vitro*,^52^ clinical trials on human volunteers have shown only modest competitive CYP2D6 and CYP3A4 inhibition with no significant clinical consequences.^53^

### Eschscholzia californica

*Eschscholzia californica* (California poppy or golden poppy) is a flowering plant of the Papaveraceae family, whose hydroalcoholic extract, rich in morphine-like isoquinoline alkaloids, is used for its sedative, analgesic, spasmolytic, and hypnotic properties.^27^ These alkaloids, such as escholtzine and allocryptopine, could play a role in the CYP3A4, CYP2C19, and CYP2C9 inhibition observed *in vitro*, while protopine could be responsible for the CYPD26 inhibition *in vitro* reported in the same study.^54^ However, no significant drug interactions have been reported in humans.

### Griffonia simplicifolia

*Griffonia simplicifolia* (*Bandiraea simplicifolia* or African bean) is a West and Central African native woody climbing shrub of the Fabaceae family, with greenish flowers and black pods. The plant seeds are used as botanicals because they contain 5-hydroxytryptophan, a serotonin precursor, with consequent antidepressant properties.^55^ Our search did not find interactions of this substance with the P450 cytochrome system.

### Lavandula angustifolia

*Lavandula angustifolia*, also called English lavender, is a flowering plant native to the Mediterranean, belonging to the Lamiaceae family. The essential oil extracted from fresh flowers, extracts derived from flowers collected just before opening and dried, fresh flowers, and dried flowers are used as botanicals. The main compounds found in these botanicals are terpenes, such as linalool, tannins, and hydroxycoumarins. English lavender has traditionally been used as an antimicrobic or as an adjuvant in mood disorders, such as restlessness or insomnia.^27^ While an *in vivo* study on rats showed CYP2D6 inhibition and CYP2A induction,^56^ a small *in vivo* study on humans did not report significant P450 cytochrome interactions after the administration of an oral extract of lavender for 11 days.^57^

### Malpighia punicifolia

*Malpighia punicifolia* var. *lancifolia* (*Malpighia emarginata* or acerola) is a Central and Southern American native evergreen shrub or small tree with bright red fruits 1–3 cm long. These fruits are often used as botanicals for their high antioxidant content and are traditionally administered for treatment of upper respiratory symptoms.^55^ Our search has not found reports of significant drug interactions associated with this botanical.

### Matricaria chamomilla

*Matricaria chamomilla* (*Matricaria recutita*, German chamomile or wild chamomile) of the Asteraceae family, an annual plant, whose structure and properties are very similar to *C. nobile*, has also traditionally been used as an infusion (chamomile tea) or as an extract for its relaxing properties and as a sleep adjuvant.^25^ Its derivatives contain apigenine, a natural flavonoid with antispasmodic effects, which could also be responsible for the observed *in vitro* inhibition of CYP3A4 and CYP1A1.^58^ Chamomile tea has also shown *in vivo* CYP1A2 inhibition capability in rats.^34^ The crude essential oil shows moderate *in vitro* inhibition of CYP2C9, CYP2D6, and CYP3A4 and strong inhibition of the CYP21A2 isoform.^59^ The chamomile leaf extract has also shown *in vitro* CYP3A4 inhibition properties.^49^ CYP3A4 inhibition was also observed in a case report on a subject [in a group of 300] who drank chamomile tea regularly while consuming cyclosporine for a renal transplant and showed consequent elevated levels of the immunosuppressant.^60^ Although limited data are available, caution should be exercised with regard to the consumption of CYP3A4-metabolized drugs with chamomile tea.

### Melaleuca alternifolia

*Melaleuca alternifolia* (tea tree) is a small tree of the Myrtaceae family, native of Australia. Its essential oil has traditionally been used for the treatment of upper respiratory symptoms.^25^ Caution should be exercised if the botanical is assumed regularly because one of its main constituents, p-cymene, has shown broad competitive *in vitro* inhibition of the P450 cytochrome system.^61^ However, widespread use of this product with no reported consequences casts doubt on the real clinical significance of these interactions.

### Melissa officinalis

*Melissa officinalis* (Balm mint or lemon balm), native to South-Central Europe, is a perennial herbaceous plant belonging to the Lamiaceae family. Its leaves are commonly used in teas and their extract is commonly used as a botanical for its properties as a sleep and digestive aid.^25^ Our search did not find significant drug interactions for *M. officinalis* derivatives.

### Ocimum tenuiflorum

*Ocimum tenuiflorum*, commonly known as holy basil, is an aromatic plant in the Lamiaceae family. Extracts from its leaves are used as botanicals, often as essential oils. The main substance contained in the essential oil is the guaiacol eugenol, which has antiseptic properties.^27^ While an *in vivo* study on rats showed a potential inhibition of CYP3A1 and CYP3A2,^62^ studies on drug interactions involving humans have yet to be conducted; therefore, the real clinical significance of the animal study remains questionable.

### Olea europaea

*Olea europaea* (olive or European olive) of the Oleaceae family, although native to the Mediterranean area, is a tree that is grown all over the world. Its fruits are commonly used as food or for olive oil production, while its leaves are a source of essential oil, or subjected to extraction of derivatives, such as oleuropein and hydroxytyrosol, which have shown preclinical neuroprotective properties.^27^ Oleuropein is also thought to be responsible for *in vitro* CYP1A2 and CYP3A4 inhibition properties^63,64^ and *in vivo* CYP2E1 induction capability observed in rats.^65^ Maslinic acid, a compound derived from the olive fruit, has also shown broad *in vivo* (in rats) and *in vitro* P450 cytochrome system inhibition properties.^66^ However, since olive oil and olive fruits are commonly used in the human diet, significant drug interactions seem unlikely.

### Panax ginseng

*Panax ginseng*, belonging to the Arialaceae family, is a species of plant whose root is used in foods and beverages. It is also used as a botanical medicine to boost memory or to treat fatigue, menopause symptoms, and diabetes.^25^ Ginsenosides, the major active constituents of the root, have been studied for their antioxidants and antiproliferative effects.^67^ Drug interaction studies conducted *in vitro* have reported different results, yielding inconclusive data and suggesting only a weak interaction with the P450 cytochrome system, with a modest inhibition of CYP3A4, CYP1A2, CYP2C9, CYP2C19, and CYP2D6. However, two studies conducted in healthy human volunteers using a midazolam probe found significant CYP3A induction; therefore, it is advised that people taking CYP3A4 substrates with narrow therapeutic indices, such as cyclosporine, tacrolimus, irinotecan, and sirolimus, should avoid coadministration of *P. ginseng*, as they could be at risk of therapeutic failure.

Two case reports involving *P. ginseng* and warfarin coadministration are described in the literature, with an observed reduction in therapeutic action; overall adverse drug effects with anticoagulants appear to be rare, as CYP2C9, one of the two P450 cytochromes involved in warfarin metabolism, is not affected by *P. ginseng*. However, close clinical monitoring is still suggested for patients taking warfarin with *P. ginseng*.^23,68^ For *P. ginseng* root extract, CYP3A induction and CYP2D inhibition were described in an *in vivo* animal model^7,8,69^; Anderson *et al.* described no clinically significant effects for CYP3A in a human *in vivo* model.^70^ Numerous potential interactions are described in *in vitro* study models, but no clinically proven effects have been found. The widespread use of this botanical suggests that it may have no clinically significant effects as Anderson *et al.* found in their investigation.

### Passiflora incarnata

*Passiflora incarnata*, also called purple passionflower, is a perennial vine native to the Southeastern United States belonging to the Passifloraceae family. Traditionally, it has been used for the treatment of neuralgia, generalized seizures, hysteria, nervous tachycardia, spasmodic asthma, spasmodic visceral symptoms, and insomnia.^71^ Despite the absence of data on possible interactions between passionflower consumption and cytochromes, two case reports provide data that deserve to be taken into consideration regarding *P. incarnata* consumption. Fisher *et al.* reported the case of a 34-year-old female patient who developed severe nausea, vomiting, drowsiness, prolonged QTc, and episodes of no sustained ventricular tachycardia following self-administration of *P. incarnata* at therapeutic doses. Her condition required hospitalization for cardiac monitoring and intravenous fluid therapy. The authors hypothesized that this patient may have had polymorphic variation of xenobiotic metabolizing genes such as CYP2D6.^71^

Carrasco *et al.* reported the case of a 40-year-old male patient being treated with lorazepam for generalized anxiety disorder. The patient's self-medication with *Valeriana officinalis* and *P. incarnata* was correlated with the onset of shaking hands, dizziness, throbbing and muscular fatigue (family medical history ruled out essential tremor, Parkinson's disease, Wilson's disease, and other symptom-related pathologies).^72^ Since there are only a very limited number of cases of rare episodes, it is difficult to evaluate the real clinical impact of *P. incarnata* consumption; however, pending further studies, it is recommended that an accurate medical history be taken before evaluating its use, especially at full dosage.

### Polygonum cuspidatum

*Polygonum cuspidatum*, also called Asian or Japanese knotweed, is an herbaceous perennial plant of the Polygonaceae family native to East Asia, Japan, China, and Korea and subsequently exported to North America and Europe. It is generally used for its content of resveratrol, a polyphenol widely used for its anti-inflammatory, antioxidant, and immunomodulator potential.^55^ Resveratrol has also been used in clinical trials to test its antitumoral activity. One of the possible underlying mechanism of its action is P450 cytochrome inhibition, with consequent reduced cell exposure to activated carcinogens. In fact, resveratrol has shown *in vivo* and *in vitro* CYP3A4 inhibition capabilities, even in humans. Chow *et al*.^73^ observed an increase in buspirone blood levels after coadministration with resveratrol in a clinical trial. The same authors observed a weak *in vivo* CYP1A2 induction in a study, in which healthy volunteers were administered 1 g of resveratrol per day for 4 weeks. The ratio between caffeine and the metabolite, paraxanthine, was then measured. A modest decrease in CYP2D6 and CYP2C9 activity was also observed in the same study.^74^ In addition, Chi *et al.*, using an *in vivo* animal model, found that the decoction from the crude drug significantly increased systemic exposure and brain concentration of Carbamazepine, likely through the inhibition of CYP3A4 activities.^75^ Further clinical trials are required to confirm these interactions; however, caution should be exercised in the concomitant administration of *P. cuspidatum* with CYP3A4 metabolized drugs such as carbamazepine.

### Rhodiola rosea

*Rhodiola rosea* (whose common names include golden root, rose root, roseroot, Arctic root, lignum rhodium, and orpin rose) is a perennial flowering plant native of wild Arctic regions of Europe, Asia, and North America. *R. rosea* has been used as an adaptogen and nutraceutical for the treatment of minor anxiety and depression.^55^ Two of its isolable components, Rhodiosin and Rhodionin, were identified by Xu *et al.* as *in vitro* inhibitors of CYP2D6,^76^ while Hellum *et al*. identified the rhizome extract as capable of *in vitro* CYP3A4 inhibition.^77^ Spanakis *et al.* reported that *R. rosea* significantly alters the pharmacokinetic properties of losartan after concurrent oral administration in an *in vivo* rabbit model.^78^ Maniscalco *et al.* reported the case of a 68-year-old female patient with recurrent moderate depressive disorder with somatic syndrome being treated with paroxetine. The patient developed a serotonergic syndrome with vegetative symptoms, a feeling of restlessness and trembling after she began taking a nonspecified formulation of *R. rosea*.^79^ Thu *et al.* identified *R. rosea* rhizome extract as an *in vitro* and *in vivo* CYP2C9 inhibitor in a human model, excluding *in vivo* inhibition of CYP3A4, CYP2D6, and CYP1A2.^80–82^ In light of these findings, considerable caution should be exercised in the concomitant administration of *R. rosea* with drugs metabolized by CYP2C9.

### Salvia officinalis

*Salvia officinalis*, also called sage, is a perennial evergreen subshrub native to the Mediterranean region. The plant is a member of the Lamiaceae family. The medicinal parts of the plant comprise the fresh leaves and the fresh flowering aerial parts, the dried leaves, and the oils extracted from the flowers and stems. Extracts contain flavonoids, thujones, camphor, and rosmarinic acid. *S. officinalis* is traditionally used to treat upper respiratory symptoms and as an antiseptic.^27^ In one study, a 5-week sage infusion in rats was associated with upregulation of CYP2E1^83^; however, the absence of clinical studies on humans and the widespread use of *S. officinalis*, also as a food, cast doubt on the real clinical significance of this study.

### Sambucus nigra

*Sambucus nigra*, commonly called elderberry, is a species of flowering plants native to Europe and North America belonging to the Adoxaceae family.^25^ *S. nigra* berry extract has been described in only a very limited number of *in vitro* studies as a very weak inhibitor of CYP1A2, CYP2D6, and CYP3A4^84,85^ (also in association with *E. purpurea*); hence, the true clinical significance of this action appears dubious.

### Uncaria tomentosa

*Uncaria tomentosa*, commonly called cat's claw (due to the claw-shaped thorns), is a vine native to the tropical areas of Central and South America belonging to the Rubiaceae family. The bark is used as a nutraceutical in support of the body's natural defences and joint function.^25^ *U. tomentosa bark* extract has been identified *in vitro* as a potent CYP3A4 inhibitor in human cell lines.^86,87^ Quílez *et al.*, using an *in vivo* mouse model, reported an increase in the effects attributable to diazepam, a drug metabolized mainly by CYP3A4.^88^ López Galera *et al.* reported the case, later cited by Müller and Kanfer, of an HIV-positive female patient with hepatitis C-related cirrhosis, who had a C.min of the protease inhibitors 1.5- and 3- and a more than 10-fold higher than the recommended target (C.min for atazanavir, ritonavir, and saquinavir, respectively); these values normalized after withdrawal of *U. tomentosa*.^89,90^ Although this is an isolated case report, involving a severely physically compromised patient on polytherapy, these data suggest that caution should exercised in the administration of *U. tomentosa* with drugs metabolized by CYP3A4, at least in conditions of polytherapy and/or hepatic impairment.

### Valeriana officinalis

*V. officinalis*, belonging to the Caprifoliaceae family, is a perennial flowering plant native to Europe and Asia traditionally used for anxiolytic and sedative purposes.^25^ Valeric acid, which is believed to be responsible for its activity, has been identified *in vitro* to interact with CYP3A4, CYP2C9, and CYP2C19. For *V. officinalis* root extract, there are reports of *in vitro* interactions with CYP3A4 (also *in vivo* in a murine model), CYP3A5, CYP219, and, with a dynamic dose-dependent mechanism, both induction and modest inhibition of CYP2D6.^91,92^ Donovan *et al.*^92^ reported a very modest inhibition of CYP2D6 and CYP3A4 in an *in vivo* human model, concluding that typical doses are unlikely to produce clinically significant effects. Kelber *et al.* reported the absence of clinically relevant effects *in vivo* in a review.^93^ Regarding both the case reports and the articles on reported or potential interactions, drugs active on the central nervous system, such as lorazepam and haloperidol, could account for the development of neurological symptoms, likely attributable to an additive effect.^94–98^

### An unexpected scenario

Our analysis, contrary to our original expectations, did not reveal any products developed specifically to counteract the current pandemic. Perhaps we should have extended our analysis to include a greater number of nutraceuticals. In fact, one of the promising therapeutic strategies against COVID-19 virus infection are the enzyme inhibitors found in natural compounds. Thoroughly investigated using molecular docking, natural compounds, such as the flavonoid class, constitute a possible target to identify antiviral drugs, thanks to their wide range of biological properties (antioxidant, anti-inflammatory, and antiviral activities).^99^ This class of secondary metabolites is found abundantly in a variety of medicinal plants, fruits, and vegetables. Indeed, onions, kale, lettuce, tomatoes, apples, grapes, and berries are considered to be rich sources of flavonoids.^100^ Of the flavonoids, flavonols constitute the most studied compounds against coronavirus.^101^ Indeed, kaempferol, quercetin, and myricetin, along with their derivatives, are likely some of the most documented molecules with antiviral activities against SARS-CoV-2. Nonetheless, in our assessment of the most widely sold botanicals, we did not find any products whose flavonol content was highlighted, or that were promoted for their possible anticoronavirus effect in terms of prevention or treatment; for example, the pulp of the fruit of *P. incarnata* contains on average 158.0 mg/mL of total flavonoids, 16.2 mg/mL of isoorientin, and 0.42 mg/g of quercetin^102^; similarly *S. nigra* contains flavonoids such as quercetin and anthocyanidins^103^; and *P. cuspidatum* contains flavonoids such as quercetin and catechin.^104^

## DISCUSSION

In this study, we focused on the analysis of possible interactions strictly related to the action of cytochromes, excluding from our analysis known mechanisms of action of the investigated substances, which are not directly related to cytochromes. Future studies examining these mechanisms could further enrich our knowledge of the safety of these substances. Furthermore, the actual effectiveness of each botanical, taken individually or in combination, was not evaluated, since as market data suggest, botanicals are assumed regardless of their demonstrated effectiveness, and it is therefore possible that potential interactions may occur. Another limitation of the present study is its focus on drug interactions involving only the P450 cytochrome system, while other significant interactions have been recognized in the literature, for example, for *P. nigrum*, where p-glycoprotein may play an important role in elevated drug blood levels as a consequence of enhanced absorption.^105^

In conclusion, our analysis of the data on the investigated plant species reveals a highly complex constellation of potential effects deriving from their use as extracts or as derived substances. Although several studies point to potential complex interactions, clinical evidence remains decidedly limited; in fact, of the 28 sources examined, only 2 (*C. paradisi* and *R. rosea*) show *in vivo* pharmacological interactions in humans. For the other botanicals that were examined, there is weak evidence of interactions or only potential interactions shown *in vitro* or in animal models and a lack of clinical drug interaction reports; therefore, these findings are of dubious clinical significance. Clearly, clinicians should exclude products with proven interactions, and this review provides them with an additional tool to make decisions based on a rational approach, evaluating the patient's whole therapeutic plan and considering his or her overall condition, therefore, prescribing substances based on a rational risk-benefit ratio. Finally, this study provides a rational assessment of the most used nutraceuticals, including popular self-medication products, to simplify patient management even in complex health care situations such as the current pandemic.

## Supplementary Material

Supplemental data

Supplemental data
