# Supplementary material for: A Practical Perspective on the Use of Botanicals During the COVID-19 Pandemic: From Proven to Potential Interactions
Source: J Med Food. 2022 Jan 13;25(1):1–11. doi: 10.1089/jmf.2021.0062 (PMC8787711; doi:10.1089/jmf.2021.0062)
Supplement: Supplemental data [file Supp_TableS1.docx]

| **BOTANICALS IN CALM + SLEEPERS PRODUCTS** |
| --- |
| *Valeriana officinalis (L.)* |
| *Matricaria chamomilla (L.)* |
| *Lavandula angustifolia (Mill.)* |
| *Passiflora incarnata (L.)* |
| *Escoltzia Chalifornica (Cham.)* |
| *Melissa officinalis (L.)* |
| *Camelia Sinensis (L.)* |
| *Crataegus oxyacantha (L.)* |
| *Crataegus monogyna (J.)* |
| *Griffonia simplicifolia (D.C.)* |
| *Chamaemelum nobile (L.)* |
| **BOTANICALS IN POLIVITAMINIC / MINERALS PRODUCTS** |
| *Panax ginseng (Mey.)* |
| *Eleuterococcus senticosus (Rupr. & Maxim)* |
| *Olea europea (L.)* |
| **BOTANICALS IN IMMUNOSTIMULANTS PRODUCTS** |
| *Carica papaya (L.)* |
| *Echinacea angustifolia (DC.)* |
| *Malpighia punicifolia (Nied)* |
| *Sambucus nigra (L.)* |
| *Polygonum cuspidatum (Siebold & Zucc.)* |
| *Echinacea purpurea (L.)* |
| *Uncaria tomentosa (D.C.)* |
| *Citrus arantium (L.)* |
| *Citrus paradisi (Macfad)* |
| *Rhodiola rosea (Britton)* |
| *Melaleuca alternifolia ((Maiden & Betche) Cheel, 1924)* |
| *Ocimum tenuiflorum (L.)* |
| *Salvia officinalis (L.)* |
| *Boswellia serrata (Triana & Planch.)* |

**Tab S1:** Botanicals contained in top ten neutraceutical products (excluding overlapping product formulas such as different formats of the same product or the same product present in multiple distribution channels) respectively sold in pharmaceutical, parapharmaceutical and hypermarket and supermarket channels in the 12 months between October 2019 and October 2020
