# Supplementary material for: A Practical Perspective on the Use of Botanicals During the COVID-19 Pandemic: From Proven to Potential Interactions
Source: J Med Food. 2022 Jan 13;25(1):1–11. doi: 10.1089/jmf.2021.0062 (PMC8787711; doi:10.1089/jmf.2021.0062)
Supplement: Supplemental data [file Supp_TableS2.docx]

| **NUTRACEUTICAL SOURCE** | **MOLECULE / EXTRACT** | **INTERRACTION** | **ACTIVE INGREDIENTS OF GREATEST INTEREST** | **STUDY MODEL** | | | | **REFERENCE** | **NOTE** |
| --- | --- | --- | --- | --- | --- | --- | --- | --- | --- |
| *Boswellia Serrata* | unspecificated extract | CYP1A2 (↓) | Fluvoxamine, Caffeine, Thiothixene, Olanzapine | in vitro |  |  |  | 27 | Boswellic acids (KBA and AKBA) show the greatest inhibitory power |
|  |  | CYP2C8 (↓) |  |  |  |  |  |  |  |
|  |  | CYP2C9 (↓) | Celecoxib, Diclofenac, Fluoxetine, Ibuprofen, Irbesartan, Naproxen, Amitriptyline, Phenytoin, S-warfarin, Gliclazide |  |  |  |  |  |  |
|  |  | CYP2C19 (↓) | Amitriptyline, Omeprazole, Phenytoin, Phenobarbital, Propranol, Diazepam, Clopidogrel |  |  |  |  |  |  |
|  |  | CYP2D6 (↓) | Carvedilol, Nortriptyline, Propranolol, Chlorpromazine, Codeine, Fluoxentina, Haloperidol, Tamoxifen, Venlafaxine |  |  |  |  |  |  |
| *Camellia sinensis* | Theine | CYP1A2 (Ø) | Fluvoxamine, Caffeine, Thiothixene, Olanzapine | In vitro |  |  | Human | 30 |  |
|  | Catechin gallate | CYP2C9 (↓) | Celecoxib, Diclofenac, Fluoxetine, Ibuprofen, Irbesartan, Naproxen, Amitriptyline, Phenytoin, S-warfarin, Gliclazide | In vitro |  |  | Human | 114 |  |
|  | Epigallocatechin Gallate | CYP3A4 (↓) | Alprazolam, Atorvastatin, Cyclosporine, Simvastatin | In vitro | In vivo |  | Human | 29-30 |  |
|  |  | CYP2B6 (Ø) |  | In vitro |  |  | Human | 108 |  |
|  |  | CYP2C8 (Ø) |  |  |  |  |  |  |  |
|  |  | CYP1A1 (↓) |  | In vitro |  |  | Animal | 110 - 115 |  |
|  |  | CYP1A2 (↓) |  | In vitro |  |  | Human | 114- 115 |  |
|  | Extract | CYP1A (↑) |  | in vitro | in vivo | Animal |  | 24 - 108 - 111 - 112 - 113 - 115 |  |
|  |  | CYP2B (↑) |  |  |  |  |  |  |  |
|  |  | CYP3A (↑) |  |  |  |  |  |  |  |
|  |  | CYP2C9 (↓) | Celecoxib, Diclofenac, Fluoxetine, Ibuprofen, Irbesartan, Naproxen, Amitriptyline, Phenytoin, S-warfarin, Gliclazide | in vitro |  |  | Human |  |  |
|  |  | CYP2D6 (↓) | Carvedilol, Nortriptyline, Propranolol, Chlorpromazine, Codeine, Fluoxentina, Haloperidol, Tamoxifen, Venlafaxine |  |  |  |  |  |  |
|  |  | CYP3A4 (↓) | Alprazolam, Atorvastatin, Cyclosporine |  |  |  |  |  |  |
|  |  | CYP1A1 (↑) |  | in vitro |  |  | Human |  |  |
|  |  | CYP1A2 (↑) | Fluvoxamine, Caffeine, Thiothixene, Olanzapine |  |  |  |  |  |  |
|  |  | CYP1A2 (↓) | Fluvoxamine, Caffeine, Thiothixene, Olanzapine |  |  |  |  |  |  |
|  |  | CYP3A4 (↓) | Alprazolam, Atorvastatin, Cyclosporine |  |  |  |  |  |  |
|  |  | CYP2B6 (Ø) |  | In vitro |  |  | Human | 108 |  |
|  |  | CYP2C8 (Ø) |  |  |  |  |  |  |  |
| *Carica papaya* | Whole Fruit Juice | CYP3A (↓) |  | In vitro |  |  | Human | 31 - 32 -33 107 |  |
|  | Whole Fruit | CYP2E1 (↓) | Acetaminophen, Ethanol |  | in vivo | Animal |  |  |  |
|  |  |  |  |  |  |  |  |  |  |
| *Chamaemelum nobile* | Extract | CYP1A2 (↓) | Fluvoxamine, Caffeine, Thiothixene, Olanzapine |  | in vivo |  |  | 35 |  |
|  |  |  |  |  |  |  |  |  |  |
|  |  |  |  |  |  |  |  |  |  |
| *Citrus arantium* | Whole Fruit Extract | CYP1A2 (↑) | Fluvoxamine, Caffeine, Thiothixene, Olanzapine |  | In vivo |  | Animal | 36-37 |  |
|  | Whole Fruit Extract | CYP3A4 (↑) | Alprazolam, Atorvastatin, Cyclosporine |  | In vivo |  | Animal |  |  |
|  |  |  |  |  |  |  |  |  |  |
| *Citrus paradisi* | Bergamottin | CYP3A4 (↓) | Alprazolam, Atorvastatin, Cyclosporine, Clopidogrel, Prasugrel | in vitro | in vivo |  | Human | 11 - 12 - 40 - 41 - 42 -43 - 44 - 45 - 46 - 118 - 120 |  |
|  |  | CYP2B6 (Ø) |  |  |  |  |  |  |  |
|  |  | CYP3A5 (Ø) |  |  |  |  |  |  |  |
|  | Paradisin - A | CYP3A4 (↓) | Alprazolam, Atorvastatin, Cyclosporine, Clopidogrel, Prasugrel |  |  |  |  |  |  |
|  | Other furanocoumarins | CYP3A4 (↓) | Alprazolam, Atorvastatin, Cyclosporine, Clopidogrel, Prasugrel |  |  |  |  |  |  |
|  |  | CYP1A1 (↓) |  |  |  | Animal |  |  |  |
|  | Bergaptene | CYP3A4 (↓) | Alprazolam, Atorvastatin, Cyclosporine, Clopidogrel, Prasugrel |  |  |  |  |  |  |
|  | Whole fruit extract | CYP3A4 (↓) | Alprazolam, Atorvastatin, Cyclosporine, Clopidogrel, Prasugrel, Itraconazolo |  | in vivo |  | Human | 38-39- 40 - 43 - 116 - 117 - 118 - 119 - 121 - 122 – 123 |  |
|  |  | CYP2D6 (↓) | Carvedilol, Nortriptyline, Propranolol, Chlorpromazine, Codeine, Fluoxentina, Haloperidol, Tamoxifen, Venlafaxine |  |  |  |  |  |  |
|  |  | CYP2C9 (↓) | Celecoxib, Diclofenac, Fluoxetine, Ibuprofen, Irbesartan, Naproxen, Amitriptyline, Phenytoin, S-warfarin |  |  |  |  |  |  |
| *Crataegus axyacantha* | NO RESULT |  |  |  |  |  |  |  |  |
| *Crataegus monogyna* | NO RESULT |  |  |  |  |  |  |  |  |
| *Echinacea angustifolia* | Root Extract | CYP3A4 (↓) | Alprazolam, Atorvastatin, Cyclosporine | in vitro |  |  |  | 49 - 50 |  |
| *Echinacea purpurea* | Extract | CYP2D6 (↓) | Carvedilol, Nortriptyline, Propranolol, Chlorpromazine, Codeine, Fluoxentina, Haloperidol, Tamoxifen, Venlafaxine | in vitro |  |  |  | 45 - 51 - 52 |  |
|  |  | CYP3A4 (↓) | Alprazolam, Atorvastatin, Cyclosporine |  | in vivo |  | Human |  |  |
|  |  | CYP2C19 (↓) | Amitriptyline, Omeprazole, Phenytoin, Phenobarbital, Propranol, Diazepam, Clopidogrel |  |  |  |  |  |  |
|  |  | CYP2C9 (↓) | Celecoxib,Diclofenac,Fluoxetina,Ibuprofene,Irbesartan,Naproxene,Amitriptilina,Fenitoina, S-warfarin, Gliclazide |  |  |  |  |  |  |
|  |  | CYP1A2 (↓) | Fluvoxamine, Caffeine, Thiothixene, Olanzapine |  | in vivo | Animal | Human |  |  |
|  |  | CYP3A1 (↓) |  |  | in vivo | Animal | Human |  |  |
|  |  | CYP3A2 (↓) |  |  | in vivo | Animal | Human |  |  |
|  |  | CYP1A1 (↑) |  |  | in vivo | Animal |  |  |  |
|  |  | CYP2D1 (↑) |  |  | in vivo | Animal |  |  |  |
| *Eleutherococcus senticosus* | Root Extract | CYP2C9 (↑) | Celecoxib,Diclofenac,Fluoxetina,Ibuprofene,Irbesartan,Naproxene,Amitriptilina,Fenitoina, S-warfarin, Gliclazide |  | in vivo | animal |  | 53 - 54 | No significant inhibition or induction effect on CYP2D6 and CYP3A4 |
|  | Root Extract | CYP2D6 (Ø) | Carvedilol, Nortriptyline, Propranolol, Chlorpromazine, Codeine, Fluoxentina, Haloperidol, Tamoxifen, Venlafaxine |  | in vivo |  | human |  |  |
|  | Root Extract | CYP3A4 (Ø) | Alprazolam, Atorvastatin, Cyclosporine |  | in vivo |  | human |  |  |
| *Escoltzia californica* | Unspecificated EtOh Extract | CYP3A4 (↓) | Alprazolam, Atorvastatin, Cyclosporine | in vitro |  |  |  | 55 | EtOh extract in vitro shows reversible inhibition of CYP2D6, due to protopine and allocryptopine. Other alkaloides, such as escholtzine and allocryptopine, are  are responsible for the inhibitory effects on CYP3A4, CYP2C9 and CYP2C19. |
|  | Unspecificated EtOh Extract | CYP2C9 (↓) | Celecoxib,Diclofenac,Fluoxetina,Ibuprofene,Irbesartan,Naproxene,Amitriptilina,Fenitoina, S-warfarin, Gliclazide |  |  |  |  |  |  |
|  | Unspecificated EtOh Extract | CYP2C19 (↓) | Amitriptyline, Omeprazole, Phenytoin, Phenobarbital, Propranol, Diazepam, Clopidogrel |  |  |  |  |  |  |
|  | Unspecificated EtOh Extract | CYP2D6 (↓↑) | Carvedilol, Nortriptyline, Propranolol, Chlorpromazine, Codeine, Fluoxentina, Haloperidol, Tamoxifen, Venlafaxine |  |  |  |  |  |  |
| *Griffonia simplicifolia* | NO RESULT |  |  |  |  |  |  |  |  |
| *Lavandula angustifolia* | unspecificated extract (essential oil) | CYP2A (↑) |  | in vivo | rat |  |  | 57 |  |
|  |  | CYP2D6 (↓) | Carvedilol, Nortriptyline, Propranolol, Chlorpromazine, Codeine, Fluoxentina, Haloperidol, Tamoxifen, Venlafaxine |  |  |  |  |  |  |
|  | lavender oil preparation silexan | CYP1A2 (Ø) | Fluvoxamine, Caffeine, Thiothixene, Olanzapine | in vivo |  |  | human | 58 | Silexan (160 mg/day) administration has no clinically relevant inhibitory or inducing effects on the CYP1A2, 2C9, 2C19, 2D6, and 3A4 enzymes in vivo. |
|  | Unspecificated extract ( lavender oil preparation silexan) | CYP2C9 (Ø) | Celecoxib, Diclofenac, Fluoxetine, Ibuprofen, Irbesartan, Naproxen, Amitriptyline, Phenytoin, S-warfarin, Gliclazide |  |  |  |  |  |  |
|  |  | CYP2C19 (Ø) | Amitriptyline, Omeprazole, Phenytoin, Phenobarbital, Propranol, Diazepam, Clopidogrel |  |  |  |  |  |  |
|  |  | CYP2D6 (Ø) | Carvedilol, Nortriptyline, Propranolol, Chlorpromazine, Codeine, Fluoxentina, Haloperidol, Tamoxifen, Venlafaxine |  |  |  |  |  |  |
|  |  | CYP3A4(Ø) | Alprazolam, Atorvastatin, Cyclosporine |  |  |  |  |  |  |
| *Malpighia punicifolia* | NO RESULT |  |  |  |  |  |  |  |  |
| *Matricaria chamomilla* | Apigenin, | CYP1A1 (↓) |  | in vitro |  |  |  | 35, 50, 59, 60, 61 | Note 1: Probable effect in humans: a potential inhibition of CYP3A4 caused by chamomile tea was seen in one patient (in 300 ). Note 2: Crude essential oil shows moderate inhibition in vitro of CYP2C9, CYP2D6 and CYP3A4 and strong inhibition of CYP21A2 isoform. This inhibitory effect is due to its mentioned components . |
|  | Apigenin, Chamazulene, trans-spiroether, cis-spiroether | CYP1A2 (↓) | Fluvoxamine, Caffeine, Thiothixene, Olanzapine |  |  |  |  |  |  |
|  | Apigenin, Chamazulene, trans-spiroether, cis-spiroether and unspecificated extract | CYP3A4 (↓) | Alprazolam, Atorvastatin, Cyclosporine |  | in vivo |  | human |  |  |
|  | Erbal Preparation ("tea" or infusion of chamomile flowers) and crude essential oil | CYP1A2 (↓) | Fluvoxamine, Caffeine, Thiothixene, Olanzapine |  | in vivo | animal |  |  |  |
|  | Apigenin, Chamazulene, trans-spiroether, cis-spiroether | CYP2C9 (↓) | Celecoxib,Diclofenac,Fluoxetina,Ibuprofene,Irbesartan,Naproxene,Amitriptilina,Fenitoina, S-warfarin, Gliclazide | In vitro |  |  |  |  |  |
|  | Apigenin, Chamazulene, trans-spiroether, cis-spiroether | CYP2D6 (↓) | Carvedilol, Nortriptyline, Propranolol, Chlorpromazine, Codeine, Fluoxentina, Haloperidol, Tamoxifen, Venlafaxine |  |  |  |  |  |  |
| *Melaleuca alternifolia* | p-cymene | CYP1A1 (Ø) |  | in vitro |  |  |  | 62 |  |
|  |  | CYP1A2 (Ø) | Fluvoxamine, Caffeine, Thiothixene, Olanzapine |  |  |  |  |  |  |
|  |  | CYP2A6 (Ø) |  |  |  |  |  |  |  |
|  |  | CYP2B6 (Ø) |  |  |  |  |  |  |  |
|  |  | CYP2C8 (Ø) |  |  |  |  |  |  |  |
|  |  | CYP2C9 (Ø) | Celecoxib, Diclofenac, Fluoxetine, Ibuprofen, Irbesartan, Naproxen, Amitriptyline, Phenytoin, S-warfarin, Gliclazide |  |  |  |  |  |  |
|  |  | CYP2C19 (Ø) | Amitriptyline, Omeprazole, Phenytoin, Phenobarbital, Propranol, Diazepam, Clopidogrel |  |  |  |  |  |  |
|  |  | CYP2D6 (Ø) | Carvedilol, Nortriptyline, Propranolol, Chlorpromazine, Codeine, Fluoxentina, Haloperidol, Tamoxifen, Venlafaxine |  |  |  |  |  |  |
|  |  | CYP2E1 (Ø) | Acetaminophen, Ethanol |  |  |  |  |  |  |
|  |  | CYP3A4 (Ø) | Alprazolam, Atorvastatin, Cyclosporine |  |  |  |  |  |  |
|  |  | CYP3A5 (Ø) |  |  |  |  |  |  |  |
|  |  | CYP4A11 (Ø) |  |  |  |  |  |  |  |
| *Melissa officinalis* | NO RESULT |  |  |  |  |  |  |  |  |
| *Ocimum tenuiflorum* | unspecificated extract | CYP3A1 (↓) |  |  | in vivo | animal |  | 63 | Polyherbal formulations (PHF) of Allium sativum L., Eugenia jambolana Lam., Momordica charantia L., Ocimum sanctum Linn., and Psidium guajava L |
|  |  | CYP3A2(↓) |  |  |  |  |  |  |  |
| *Olea europea* | Oleuropein | CYP1A2 (↓) | Fluvoxamine, Caffeine, Thiothixene, Olanzapinein v | in vitro |  |  |  | 64, 65 | A unidentified metabolide derived from oxidation of oleuropeine can inactivate CYP3A4 |
|  |  | CYP3A4 (↓) | Alprazolam, Atorvastatin, Cyclosporine |  |  |  |  |  |  |
|  |  | CYP2E1 (↑) | Acetaminophen, Ethanol | in vivo |  | animal |  | 66 |  |
|  | Hydroxytyrosol | NO RESULTS |  |  |  |  |  |  |  |
|  | Maslinic Acid (fruit) | CYP2C11 (↓) |  |  | in vivo | animal |  | 67 |  |
|  |  | CYP2E1 (↓) | Acetaminophen, Ethanol |  |  |  |  |  |  |
|  |  | CYP3AD (↓) |  |  |  |  |  |  |  |
|  |  | CYP3A2 (↓) |  |  |  |  |  |  |  |
|  |  | CYP3A4 (↓) | Alprazolam, Atorvastatin, Cyclosporine | in vitro |  |  |  |  | *inhibition in vitro on human liver microsomes |
| *Panax ginseng* | Ginsenoside Rd | CYP2C9 (↓) | Celecoxib, Diclofenac, Fluoxetine, Ibuprofen, Irbesartan, Naproxen, Amitriptyline, Phenytoin, S-warfarin, Gliclazide | in vitro |  |  | Human | 7 - 24 - 69 - 70 - 102 - 124 - 125- 128 |  |
|  |  | CYP2C19 (↓) | Amitriptyline, Omeprazole, Phenytoin, Phenobarbital, Propranol, Diazepam, Clopidogrel |  |  |  |  |  |  |
|  |  | CYP2D6 (↓) | Carvedilol, Nortriptyline, Propranolol, Chlorpromazine, Codeine, Fluoxentina, Haloperidol, Tamoxifen, Venlafaxine |  |  |  |  |  |  |
|  |  | CYP3A4 (↓) | Alprazolam, Atorvastatin, Cyclosporine |  | In vivo |  |  |  |  |
|  | Ginsenoside Rf-Rc | CYP2C9 (↓) | Celecoxib, Diclofenac, Fluoxetine, Ibuprofen, Irbesartan, Naproxen, Amitriptyline, Phenytoin, S-warfarin, Gliclazide | in vitro |  |  | Human |  |  |
|  | Ginsenoside Rg1 | CYP1A1(↑) |  | in vitro |  |  |  | 126 |  |
|  | Ginsenoside Rb2 | CYP1A1(↑) |  | in vitro |  |  |  |  |  |
|  | Gisenoside Rg3 | CYP1A2 (↓) | Fluvoxamine, Caffeine, Thiothixene, Olanzapine | in vitro |  |  |  | 128 | *weak inhibition of CYP2C19 and potent inhibition of CYP2C9 and CYP3A4 in vitro |
|  | Gisenoside Rg3, Gisenoside Rh2, Gisenoside CH and all sapogenin | CYP2C19 (↓) | Amitriptyline, Omeprazole, Phenytoin, Phenobarbital, Propranol, Diazepam, Clopidogrel | in vitro |  |  |  |  |  |
|  |  | CYP2C9 (↓) | Celecoxib, Diclofenac, Fluoxetine, Ibuprofen, Irbesartan, Naproxen, Amitriptyline, Phenytoin, S-warfarin, Gliclazide |  |  |  |  |  |  |
|  |  | CYP3A4 (↓) | Alprazolam, Atorvastatin, Cyclosporine |  |  |  |  |  |  |
|  | Purified Kampferol | CYP3A4 (↓) | Alprazolam, Atorvastatin, Cyclosporine |  |  |  |  |  |  |
|  | Extract | CYP1A1 (↓) |  | in vitro |  | Animal |  |  |  |
|  |  | CYP1A2 (↓) | Fluvoxamine, Caffeine, Thiothixene, Olanzapine |  | in vivo |  |  |  |  |
|  |  | CYP1B1 (↓) |  |  |  |  |  |  | 1*Mixed Erbal preparation made from P.G., Ginko and Crocus Sativus increased CYP1A2* |
|  |  | CYP2E1 (↓) | Acetaminophen, Ethanol |  |  |  |  |  |  |
|  |  | CYP2B1 (Ø) |  |  |  |  |  |  |  |
|  |  | CYP3A23 (Ø) |  |  |  |  |  |  |  |
|  |  | CYP1A1 (↓) |  | in vitro | in vivo | Animal | Human | 71 | Absence of significant interactions in vivo with CYP3A4 and absence of significant interaction with CYP enzymes in clinical trials.Nota 2 su induzione CYP2A1: 1*Mixed Erbal preparation made from P.G., Ginko and Crocus Sativus increased CYP1A2* |
|  |  | CYP1A2 (↓) | Fluvoxamine, Caffeine, Thiothixene, Olanzapine |  |  |  |  |  |  |
|  |  | CYP1A2 (↑) | Fluvoxamine, Caffeine, Thiothixene, Olanzapine |  | in vivo | animal |  |  |  |
|  |  | CYP1B1 (↓) |  |  |  |  |  |  |  |
|  |  | CYP3A (↑) |  |  | in vivo | Animal | Human | 129 |  |
|  |  | CYP2D (↓) |  |  | in vivo | Animal |  |  |  |
|  |  | CYP3A4 (↑) |  |  | in vivo |  | Human | 69 | *weak induction in healthy volunteers |
|  |  | CYP2D6 (↓) | Carvedilol, Nortriptyline, Propranolol, Chlorpromazine, Codeine, Fluoxentina, Haloperidol, Tamoxifen, Venlafaxine | in vitro | in vivo |  | human | 102 -127 | *effect IN VITRO exerted by crude extract and ginsenosides. NOTA IN VIVO (humans) the inhibition of CYP2D6 in statistically significant but the magnitude of of the effect is not clinically significant. |
|  |  | CYP2C19 (↓) | Amitriptyline, Omeprazole, Phenytoin, Phenobarbital, Propranol, Diazepam, Clopidogrel |  |  |  |  | 127 |  |
|  |  | CYP3A4 (↓) | Alprazolam, Atorvastatin, Cyclosporine |  |  |  |  |  |  |
|  |  | CYP2C9 (↓) | Celecoxib, Diclofenac, Fluoxetine, Ibuprofen, Irbesartan, Naproxen, Amitriptyline, Phenytoin, S-warfarin, Gliclazide |  |  |  |  |  |  |
| *Passiflora incarnata* | ONLY CASE REPORTS |  |  |  |  |  |  | 72 |  |
| *Polygonum cuspidatum* | Decotion from crude dug | CYP3A4 | Alprazolam, Atorvastatin, Cyclosporine |  | in vivo | Animal |  | 75 |  |
|  | Resveratrol | CYP3A4 (↓) | Alprazolam, Atorvastatin, Cyclosporine |  | in vivo |  | human | 73,74 | *weak inhibition of CYP1A2 in vivo; very weak inhibition of CYP2E1 in vivo (rats); moderate induction of CYP2B1/2 in vivo (rat) |
|  |  | CYP1A2 (↓) | Fluvoxamine, Caffeine, Thiothixene, Olanzapine | in vitro |  |  |  |  |  |
|  |  | CYP2D6 (↓) | Carvedilol, Nortriptyline, Propranolol, Chlorpromazine, Codeine, Fluoxentina, Haloperidol, Tamoxifen, Venlafaxine |  |  |  |  |  |  |
|  |  | CYP2C9 (↓) | Celecoxib, Diclofenac, Fluoxetine, Ibuprofen, Irbesartan, Naproxen, Amitriptyline, Phenytoin, S-warfarin, Gliclazide |  |  |  |  |  |  |
|  |  | CYP2E1 (↓) | Acetaminophen, Ethanol |  | In vivo | animal |  |  |  |
|  |  | CYP2B1 (↑) |  |  |  |  |  |  |  |
|  |  | CYP2B2 (↑) |  |  |  |  |  |  |  |
|  |  | CYP1A1 (↓) |  | in vitro |  |  |  |  |  |
|  |  | CYP1A2 (↓) | Fluvoxamine, Caffeine, Thiothixene, Olanzapine |  |  |  |  |  |  |
| *Rhodiola rosea* | Rhodiosin | CYP2D6 (↓) | Carvedilol, Nortriptyline, Propranolol, Chlorpromazine, Codeine, Fluoxentina, Haloperidol, Tamoxifen, Venlafaxine | in vitro |  |  | Human | 76- 77-78-79-80-81-82 |  |
|  | Rhodionin | CYP2D6 (↓) | Carvedilol, Nortriptyline, Propranolol, Chlorpromazine, Codeine, Fluoxentina, Haloperidol, Tamoxifen, Venlafaxine | in vitro |  |  | Human |  |  |
|  | Dry ryzome extract | CYP3A4 (↓) | Alprazolam, Atorvastatin, Cyclosporine | in vitro |  |  | Human |  |  |
|  |  | CYP2C9 (↓) | Celecoxib, Diclofenac, Fluoxetine, Ibuprofen, Irbesartan, Naproxen, Amitriptyline, Phenytoin, S-warfarin, Gliclazide | in vitro | in vivo |  | Human |  |  |
| *Salvia Officinalis* | unspecificated extract (sage tea) | CYP2E1 (↑) | Acetaminophen, Ethanol |  | in vivo | animal |  | 83 |  |
| *Sambucus nigra* | Berry Extract | CYP3A4 (↓) | Alprazolam, Atorvastatin, Cyclosporine | in vitro |  |  | Human | 84-85 |  |
|  |  | CYP2D6 (↓) | Carvedilol, Nortriptyline, Propranolol, Chlorpromazine, Codeine, Fluoxentina, Haloperidol, Tamoxifen, Venlafaxine |  |  |  |  |  |  |
|  |  | CYP1A2 (↓) | Fluvoxamine, Caffeine, Thiothixene, Olanzapine |  |  |  |  |  |  |
| *Uncaria tormentosa* | Extract cortex | CYP3A4 (↓) | Alprazolam, Atorvastatin, Cyclosporine | in vitro | in vivo | animale | Human | 50-86-87-88-89 |  |
|  | Preparation | CYP3A4 (↓) | Alprazolam, Atorvastatin, Cyclosporine |  | in vivo |  | Human |  |  |
| *Valeriana officinalis* | Valeric Acid | CYP3A4 (Ø) | Alprazolam, Atorvastatin, Cyclosporine | in vitro |  |  |  | 90 - 91 - 92-93-94-95-96-97-98-130-131 -132  133 | Absence of significant interactions in vivo |
|  |  | CYP2C9 (Ø) | Celecoxib, Diclofenac, Fluoxetine, Ibuprofen, Irbesartan, Naproxen, Amitriptyline, Phenytoin, S-warfarin, Gliclazide |  |  |  |  |  |  |
|  |  | CYP2C19 (Ø) | Amitriptyline, Omeprazole, Phenytoin, Phenobarbital, Propranol, Diazepam, Clopidogrel |  |  |  |  |  |  |
|  | Dry root extract | CYP2D6 (↓) (↑) | Donovan | in vitro | in vivo |  | Human |  | Both modest in vitro inhibition and dose dependent induction reported.Typical doses are unlikely to produce clinically significant effects |
|  |  | CYP3A4 (Ø) (↓) | Alprazolam, Atorvastatin, Cyclosporine | in vitro | in vivo | Animal | Human |  |  |
|  |  | CYP3A5 (Ø) |  | in vitro |  |  | Human |  |  |
|  |  | CYP2C19 (↑) (↓) |  | in vitro |  |  | Human |  |  |

**Tab S2:** Summary of the main interactions that were examined. Source: food or nutraceutical source examined, Substance: extract or molecule investigated, Interactions: cytochrome affected as (↑) Inductor, (↓) Repressor, (↔) Neutral, (Ø) Target, Active ingredients of greatest interest: most commonly used pharmacological substances in clinical practice. Study model: evaluation performed in vitro or in vivo with animal or human cells or subjects. Ref: bibliographic reference.

1. Gualano MR, Lo Moro G, Voglino G, Bert F, Siliquini R: Effects of Covid-19 lockdown on mental health and sleep disturbances in Italy. Int J Environ Res Public Health 2020;17:4779.

2. AUTHORS NOT LISTED, "Il mercato degli integratori. Driver e dinamiche di acquisto in tempo di COVID" (The supplement market. drivers and buying dynamics in COVID time) - Article in Italian - published on FederSalus On-line portal, Category: Our Market (Categoria: Il Nostro Mercato) date: 22/12/2020

https://www.federsalus.it/il-mercato-degli-integratori-driver-e-dinamiche-di-acquisto-in-tempo-di-covid/#_ftn1 (accessedJanuary 1, 2021).

3. IQVIA Institute for Human Data Science Reports - commerce data tracking – YTD October 2020 (<https://www.iqvia.com/insights/the-iqvia-institute/reports>)

4. World Health Organization. WHO Global Report on Traditional and Complementary Medicine 2019. World Health Organization, Geneva, 2019.

5. Danielson PB: The cytochrome P450 superfamily: Biochemistry, evolution and drug metabolism in humans. Curr Drug Metab 2002;3:561–597.

6. Guengerich FP: Cytochrome P-450 3A4: Regulation and role indrug metabolism. Annu Rev Pharmacol Toxicol 1999;39:1–17.

7. Etheridge AS, Black SR, Patel PR, So J, Mathews JM: An in vitro evaluation of cytochrome P450 inhibition and P-glycoprotein interaction with goldenseal, Ginkgo biloba, grape seed, milk thistle, and ginseng extracts and their constituents. Planta Med 2007;73:731–741.

8. Feltrin C, Farias IV, Sandjo LP, Reginatto FH, Simo˜es CMO: Effects of standardized medicinal plant extracts on drug metabolism mediated by CYP3A4 and CYP2D6 enzymes. Chem Res Toxicol 2020;33:2408–2419.

9. Gurley BJ, Swain A, Hubbard MA, Williams DK, Barone G,Hartsﬁeld F, Tong Y, Carrier DJ, Cheboyina S, Battu SK:Clinical assessment of CYP2D6-mediated herb-drug interactions in humans: Effects of milk thistle, black cohosh, golden seal, kava kava, St. John’s wort, and Echinacea. Mol Nutr Food Res 2008;52:755–763.

10. Hermann R, von Richter O: Clinical evidence of herbal drugs as perpetrators of pharmacokinetic drug interactions. Planta Med 2012;78:1458–1477.

11. Zhou S, Gao Y, Jiang W, Huang M, Xu A, Paxton JW: Interactions of herbs with cytochrome P450. Drug Metab Rev 2003;35:35–98.

12. He SM, Chan E, Zhou SF: ADME properties of herbal medicines in humans: Evidence, challenges and strategies. CurrPharm Des 2011;17:357–407.

13. Suroowan S, Mahomoodally MF: Herbal medicine of the 21^st^ century: A focus on the chemistry, pharmacokinetics and toxicity of ﬁve widely advocated phytotherapies. Curr Top Med Chem 2019;19:2718–2738.

14. Ulbricht C, Basch E, Weissner W, Hackman D: An evidence-based systematic review of herb and supplement interactions by the Natural Standard Research Collaboration. Expert Opin Drug Saf 2006;5:719–728.

15. Kennedy DA, Seely D: Clinically based evidence of drug–herb interactions: A systematic review. Expert Opin Drug Saf 2009; 9:79–124.

16. Shord SS, Shah K, Lukose A: Drug-botanical interactions: A review of the laboratory, animal, and human data for 8 common botanicals. Integr Cancer Ther 2009;8:208–227.

17. Izzo AA, Ernst E: Interactions between herbal medicines and prescribed drugs: An updated systematic review. Drugs 2009; 69:1777–1798.

18. Mukherjee PK, Ponnusankar S, Pandit S, Hazam PK, Ahmmed M, Mukherjee K: Botanicals as medicinal food and their effects on drug metabolizing enzymes. Food Chem Toxicol 2011;49: 3142–3153.

19. Gurley B: Pharmacokinetic herb-drug interactions (part 1): bAU16 Origins, mechanisms, and the impact of botanical dietary supplements. Planta Med 2012;78:1478–1489.

20. Gurley B, Fifer E, Gardner Z: Pharmacokinetic herb-drug interactions (part 2): Drug interactions involving popular botanical dietary supplements and their clinical relevance. Planta Med 2012;78:1490–1514.

21. Wanwimolruk S, Prachayasittikul V: Cytochrome P450 enzyme mediated herbal drug interactions (Part 1). EXCLI J 2014;13: 347–391.

22. Wanwimolruk S, Phopin K, Prachayasittikul V: Cytochrome P450 enzyme mediated herbal drug interactions (Part 2). EXCLI J 2014;13:869–896.

23. Cho HJ, Yoon IS: Pharmacokinetic interactions of herbs with cytochrome p450 and p-glycoprotein. Evid Based Complement Alternat Med 2015;2015:736431.

24. Bano G, Amla V, Raina RK, Zutshi U, Chopra CL: The effect of piperine on pharmacokinetics of phenytoin in healthy volunteers. Planta Med 1987;53:568–569.

25. World Health Organization: WHO Monographs on Selected Medicinal Plants vol 1–4. World Health Organization, Geneva, 1999–2009. Italian Phytotherapy Society (SIFIT) Edition 2002–2017.

26. Frank A, Unger M: Analysis of frankincense from various Boswellia species with inhibitory activity on human drug metabolising cytochrome P450 enzymes using liquid chromatography mass spectrometry after automated on-line extraction.J Chromatogr A 2006;1112:255–262.

27. Heber D: PDR (Physicians Desk Reference) for Herbal Medicine, 4th ed. Thomson Healthcare, Inc., Edition. 07645-1725 Montvale, Bergen County, New Jersey, United States

28. Albassam AA, Markowitz JS: An appraisal of drug-drug interactions with green tea (Camellia sinensis). Planta Med 2017; 83:496–508.

29. Oga EF, Sekine S, Shitara Y, Horie T: Pharmacokinetic herb-drug interactions: insight into mechanisms and consequences.Eur J Drug Metab Pharmacokinet 2016;41:93–108.

30. Hidaka M, Fujita KI, Ogikubo T, Yamasaki K, Iwakiri T,Okumura M, Kodama H, Arimori K: Potent inhibition by starfruit of human cytochrome P450 3A (CYP3A) activity. Drug Metab Dispos 2004;32:581–583.

31. Chatuphonprasert W, Jarukamjorn K: Impact of six fruits—Banana, guava, mangosteen, pineapple, ripe mango and ripe papaya—On murine hepatic cytochrome P450 activities. J Appl Toxicol 2012;32:994–1001.

32. Nutescu EA, Shapiro NL, Ibrahim S, West P: Warfarin and its interactions with foods, herbs and other dietary supplements. Expert Opin Drug Saf 2006;5:433–451.

33. Jia Y, Zou J, Wang Y, Zhang X, Shi Y, Liang Y, Guo D, Yang M: Action mechanism of Roman chamomile in the treatment of anxiety disorder based on network pharmacology. J Food Bio-chem 2021;45:e13547.

34. Srivastava JK, Shankar E, Gupta S: Chamomile: A herbal medicine of the past with bright future. Mol Med Rep 2010;3: 895–901.

35. Malhotra S, Bailey DG, Paine MF, Watkins PB: Seville orange juice-felodipine interaction: Comparison with dilute grapefruit juice and involvement of furocoumarins. Clin Pharmacol Ther 2001;69:14–23.

36. Zhou L, Man C, Linlin Z, Dongsheng W, Tao T, Wenbo W,Sheng W, Huiyong H, Xinjian Q: Potential metabolic drug–drug interaction of Citrus aurantium L. (Rutaceae) evaluating by its effect on 3 CYP450. Front Pharmacol 2018;9:895.

37. Khalil MNA, Farghal HH, Farag MA: Outgoing and potential trends of composition, health beneﬁts, juice production and waste management of the multi-faceted Grapefruit Citrus C paradisi: A comprehensive review for maximizing its value. Crit Rev Food Sci Nutr 2020:1–22. [Epub ahead of print]; DOI:

10.1080/10408398.2020.1830364.

38. Cristo´bal-Luna JM, A´lvarez-Gonza´lez I, Madrigal-Bujaidar E, Chamorro-Cevallos G: Grapefruit and its biomedical, antigenotoxic and chemopreventive properties. Food Chem Toxicol 2018;112:224–234.

39. Bailey DG, Dresser GK, Kreeft JH, Munoz C, Freeman DJ, Bend JR: Grapefruit-felodipine interaction: Effect of unprocessed fruit and probable active ingredients. Clin Pharmacol Ther 2000;68:468–477.

40. Goosen TC, Cillie´ D, Bailey DG, Yu C, He K, Hollenberg PF, Woster PM, Cohen L, Williams JA, Rheeders M, Dijkstra HP: Bergamottin contribution to the grapefruit juice-felodipine interaction and disposition in humans. Clin Pharmacol Ther 2004; 76:607–617.

41. Ho PC, Saville DJ, Wanwimolruk S: Inhibition of human CYP3A4 activity by grapefruit ﬂavonoids, furanocoumarins and related compounds. J Pharm Pharm Sci 2001;4:217–227.

42. Holmberg MT, Tornio A, Joutsi-Korhonen L, Neuvonen M, Neuvonen PJ, Lassila R, Niemi M, Backman JT: Grapefruit juice markedly increases the plasma concentrations and antiplatelet effects of ticagrelor in healthy subjects. Br J Clin Pharmacol 2013;75:1488–1496.

43. Lin HL, Kenaan C, Hollenberg PF: Identiﬁcation of the residue in human CYP3A4 that is covalently modiﬁed by bergamottin and the reactive intermediate that contributes to the grapefruit juice effect. Drug Metab Dispos 2012;40:998–1006.

44. Mertens-Talcott SU, Zadezensky I, De Castro WV, Derendorf H, Butterweck V: Grapefruit-drug interactions: Can interactions with drugs be avoided? J Clin Pharmacol 2006;46:1390–1416.

45. Paine MF, Criss AB, Watkins PB: Two major grapefruit juice components differ in time to onset of intestinal CYP3A4 inhibition. J Pharmacol Exp Ther 2005;312:1151–1160.

46. Brandin H, Myrberg O, Rundlo¨f T, Arvidsson AK, Brenning G: Adverse effects by artiﬁcial grapefruit seed extract products in patients on warfarin therapy. Eur J Clin Pharmacol 2007;63:565–570.

47. Tankanow R, Tamer HR, Streetman DS, Smith SG, Welton JL, Annesley T, Aaronson KD, Bleske BE: Interaction study between digoxin and a preparation of hawthorn (Crataegus oxyacantha). J Clin Pharmacol 2003;43:637–642.

48. Freeman C, Spelman K: A critical evaluation of drug interactions with Echinacea spp. Mol Nutr Food Res 2008;52:789–798.

49. Budzinski W, Foster BC, Vandenhoek’ S: Amason an in vitro evaluation of human cytochrome P450 3A4 inhibition by selected commercial herbal extracts and tinctures. Phytomedicine 2000;7:273–282.

50. Mrozikiewicz PM, Bogacz A, Karasiewicz M, Mikolajczak PL, Ozarowski M, Seremak-Mrozikiewicz A, Czerny B, Bobkiewicz-Kozlowska T, Grzeskowiak E: The effect of standardized Echinacea purpurea extract on rat cytochrome P450 expression level. Phytomedicine 2010;17:830–833.

51. Yale SH, Glurich I: Analysis of the inhibitory potential of Ginkgo biloba, Echinacea purpurea, and Serenoa repens on the metabolic activity of cytochrome P450 3A4, 2D6, and 2C9. J Altern Complement Med 2005;11:433–439.

52. Hu Z, Yang Z, Ho P, Chan SY, Heng PWS, Chan E, Duan E, Koh HL, Zhou S: Herb-drug interactions. Drugs 2005;65:1239–1282.

53. Donovan JL, DeVane CL, Chavin DL, Taylor RM, J Markowitz JS: Siberian Ginseng (Eleutheroccus senticosus) effects On CYP2D6 and CYP3A4 activity in normal volunteers. Drug Metab Dispos 2003;31:519–522.

54. Manda VK, Ibrahim MA, Dale OR, Kumarihamy M, Cutler SJ, Khan IA, Larry AW, Muhammad, Khan SI: Modulation of CYPs, P–gp, and PXR by Eschscholzia californica (California Poppy) and its alkaloids. Planta Med 2016;82:551–558.

55. Guida alle piante ﬁtoterapiche -Guide to phytotherapeutic plants (from CODIFA bAU18 database) 2019 EDRA edition.

56. Noskova’ K, Dovrteˇlova´ G, Zendulka O, Rˇemı´nek R, Jurˇica J:The effect of (-)-linalool on the metabolic activity of liver CYP enzymes in rats. Physiol Res 2016;65(Suppl 4):S499–S504.

57. Doroshyenko O, Rokitta D, Zadoyan G, Klement S, Schla¨fke S, Dienel A, Gramatte´ T, Lu¨ck H, Fuhr U: Drug cocktail interaction study on the effect of the orally administered lavender oil preparation silexan on cytochrome P450 enzymes in healthy volunteers. Drug Metab Dispos 2013;41:987–993.

58. Tang D, Chen K, Huang L, Li J: Pharmacokinetic properties and drug interactions of apigenin, a natural ﬂavone. Expert Opin Drug Metab Toxicol 2017;13:323–330.

59. Ganzera M, Schneider P, Stuppner H: Inhibitory effects of the essential oil of chamomile (Matricaria recutita L.) and its major constituents on human cytochrome P450 enzymes. Life Sci 2006;78:856–861.

60. Colombo D, Lunardon L, Bellia G: Cyclosporine and herbal supplement interactions. J Toxicol 2014;2014:145325.

61. Meesters RJ, Duisken M, Hollender J: Cytochrome P450-catalysed arene-epoxidation of the bioactive tea tree oil ingredient p-cymene: Indication for the formation of a reactive allergenic intermediate? Xenobiotica 2009;39:663–671.

62. Kumar D, Trivedi N, Dixit RK: Evaluation of the synergistic effect of Allium sativum, Eugenia jambolana, Momordica charantia, Ocimum sanctum, and Psidium guajava on hepatic and intestinal drug metabolizing enzymes in rats. J Intercult Ethnopharmacol 2016;5:372–382.

63. Stupansa I, Murrayb A, Kirlicha A, Tucka KL, Hayballa PJ: Inactivation of cytochrome P450 by the food-derived complex phenol oleuropein. Food Chem Toxicol 2001;39:1119–1124.

64. Zhoua G, Koha HL, Gaob Y, Gongc Z, Leed EJD: Herbal bioactivation: The good, the bad and the ugly. Life Sci 2004;74:935–968.

65. Potocnjak I, Skoda M, Pernjak-Pugel E, Pavletic Per M, Domitrovic R: Oral administration of oleuropein attenuates cisplatin-induced acute renal injury in mice through inhibition of ERK signaling. Mol Nutr Food Res 2016;60:530–541.

66. Sun M, Tang Y, Ding T, Liu M, Wang X: Investigation of cytochrome P450 inhibitory properties of maslinic acid, a bioactive compound from Olea europaea L., and its structure-activity relationship. Phytomedicine 2015;22:56–65.

67. Kiefer D, Pantuso T: Panax ginseng. Am Fam Physician 2003; 68:1539–1542.

68. Ramanathan MR, Penzak SR: Pharmacokinetic drug interactions with Panax ginseng. Eur J Drug Metab Pharmacokinet 2017;42:545–557.

69. Yokotani K, Chiba T, Sato Y, Nakanishi T, Murata M, UmegakiK: Effect of three herbal extracts on cytochrome P450 and possibility of interaction with drugs [in Japanese]. Shokuhin

Eiseigaku Zasshi 2013;54:56–64.

70. Anderson GD, Rosito G, Mohustsy MA, Elmer GW: Drug interaction potential of soy extract and Panax ginseng. J Clin Pharmacol 2003;43:643–648.

71. Fisher AA, Purcell P, Le Couteur DG: Toxicity of Passiﬂora incarnata L. J Toxicol Clin Toxicol 2000;38:63–66.

72. Carrasco MC, Vallejo JR, Pardo-de-Santayana M, Peral D,Martı´n MA, Altimiras J: Interactions of Valeriana ofﬁcinalis L. and Passiﬂora incarnata L. in a patient treated with lorazepam. Phytother Res 2009;23:1795–1796.

73. Chow HH, Garland LL, Hsu CH, Vining DR, Chew WM, Miller JA, Perloff M, Crowell JA, Alberts DS: Resveratrol modulates drug- and carcinogen-metabolizing enzymes in a healthy volunteer study. C*ancer Prev Res (Phila)* 2010;3:1168-1175.

74. Detampel P, Beck M, Kra¨henbu¨hl S, Huwyler J: Drug interaction potential of resveratrol. Drug Metab Rev 2012;44:253–265.

75. Chi YC, Lin SP, Hou YC: A new herb-drug interaction of Polygonum cuspidatum, a resveratrol-rich nutraceutical, with carbamazepine in rats. Toxicol Appl Pharmacol 2012;263:315–322.

76. Xu W, Zhang T, Wang Z, Liu T, Liu Y, Cao Z, Sui Z: Two potent cytochrome P450 2D6 inhibitors found in Rhodiola rosea. Pharmazie 2013;68:974–976.

77. Hellum BH, Tosse A, Hoybakk K, Thomsen M, Rohloff J,Georg Nilsen O: Potent in vitro inhibition of CYP3A4 and P-glycoprotein by Rhodiola rosea. Planta Med 2010;76:331–338.

78. Spanakis M, Vizirianakis IS, Batzias G, Niopas I: Pharmacokinetic interaction between losartan and Rhodiola rosea in rabbits. Pharmacology 2013;91:112–116.

79. Maniscalco I, Toffol E, Giupponi G, Conca A: The interaction of Rhodiola rosea and antidepressants. A case report [in German]. Neuropsychiatr 2015;29:36–38.

80. Thu OKF, Spigset O, Hellum B: Noncompetitive inhibition of human CYP2C9 in vitro by a commercial Rhodiola rosea product. Pharmacol Res Perspect 2017;5:e00324.

81. Thu OK, Spigset O, Nilsen OG, Hellum B: Effect of commercial Rhodiola rosea on CYP enzyme activity in humans. Eur J Clin Pharmacol 2016;72:295–300.

82. Thu OK, Nilsen OG, Hellum B: In vitro inhibition of cytochrome P-450 activities and quantiﬁcation of constituents in a selection of commercial Rhodiola rosea products. Pharm Biol2016;54:3249–3256.

83. Lima CF, Fernandes-Ferreira M, Pereira-Wilson C: Drinking of Salvia ofﬁcinalis tea increases CCl(4)-induced hepatotoxicity in mice. Food Chem Toxicol 2007;45:456–464.

84. Schrøder-Aasen T, Molden G, Nilsen OG: In vitro inhibition of CYP3A4 by the multiherbal commercial product Sambucus Force and its main constituents Echinacea purpurea and Sambucus nigra. Phytother Res 2012;26:1606–1613.

85. Langhammer AJ, Nilsen OG: In vitro inhibition of human CYP1A2, CYP2D6, and CYP3A4 by six herbs commonly used in pregnancy. Phytother Res 2014;28:603–610.

86. Sato Y, Sasaki T, Takahashi S, Kumagai T, Nagata K: Development of a highly reproducible system to evaluate inhibition of cytochrome P450 3A4 activity by natural medicines. J Pharm Pharm Sci 2015;18:316–327.

87. Quı´lez AM, Saenz MT, Garcı´a MD: Uncaria tomentosa (Willd.ex. Roem. & Schult.) DC. and Eucalyptus globulus Labill. interactions when administered with diazepam. Phytother Res 2012;26:458–461.

88. Lo´pez Galera RM, Ribera Pascuet E, Esteban Mur JI, Montoro Ronsano JB, Jua´rez Gime´nez JC: Interaction between cat’s claw and protease inhibitors atazanavir, ritonavir and saquinavir. Eur J Clin Pharmacol 2008;64:1235–1236.

89. Mu¨ller AC, Kanfer I: Potential pharmacokinetic interactions between antiretrovirals and medicinal plants used as complementary and African traditional medicines. Biopharm Drug Dispos 2011;32:458–470.

90. Hellum BH, Hu Z, Nilsen OG: The induction of CYP1A2, CYP2D6 and CYP3A4 by six trade herbal products in cultured primary human hepatocytes. Basic Clin Pharmacol Toxicol 2007;100:23–30.

91. Zhou S, Chan E, Li SC, Huang M, Chen X, Li X, Zhang Q, Paxton JW: An approach to the in vitro evaluation of potential for cytochrome P450 enzyme inhibition from herbals and other natural remedies. Phytomedicine 2004;11:98–104.

92. Donovan JL, DeVane CL, Chavin KD, Wang JS, Gibson BB, Gefroh HA, Markowitz JS: Multiple night-time doses of valerian (Valeriana ofﬁcinalis) had minimal effects on CYP3A4 activity and no effect on CYP2D6 activity in healthy volunteers.Drug Metab Dispos 2004;32:1333–1336.

93. Kelber O, Nieber K, Kraft K: Valerian: No evidence for clinically relevant interactions. Evid Based Complement Alternat Med 2014;2014:879396.

94. Dalla Corte CL, Fachinetto R, Colle D, Pereira RP, Avila DS,Villarinho JG, Wagner C, Pereira ME, Nogueira CW, Soares FA, Rocha JB: Potentially adverse interactions between haloperidol and valerian. Food Chem Toxicol 2008;46:2369–2375.

95. Hoban CL, Byard RW, Musgrave IF: Analysis of spontaneous adverse drug reactions to echinacea, valerian, black cohosh and ginkgo in Australia from 2000 to 2015. J Integr Med 2019;17: 338–343.

96. Wilson V, Maulik SK: Herb-drug interactions in neurological disorders: A critical appraisal. Curr Drug Metab 2018;19:443–453.

97. Lefebvre T, Foster BC, Drouin CE, Krantis A, Livesey JF, Jordan SA: In vitro activity of commercial valerian root extracts against human cytochrome P450 3A4. J Pharm Pharm Sci2004;7:265–273.

98. Lalani S, Poh CL: Flavonoids as antiviral agents for enterovirus A71 (EV-A71). Viruses 2020;12:712.

99. Mouffouk C, Mouffouk S, Mouffouk S, Hambaba L, Haba H: Flavonols as potential antiviral drugs targeting SARS-CoV-2 proteases (3CLpro and PLpro), spike protein, RNA-dependent RNA polymerase (RdRp) and angiotensin-converting enzyme II receptor (ACE2). Eur J Pharmacol 2021;891:173759.

100. Gorla US, Rao GK, Kulandaivelu US, Alavala RR, Panda SP: Lead ﬁnding from selected ﬂavonoids with antiviral (SARS-CoV-2) potentials against COVID-19: An in-silico evaluation. Comb Chem High Throughput Screen 2021;24:879–890.

101. Gurley BJ, Gardner SF, Hubbard MA, Williams DK, Gentry WB, Cui Y, Ang CY: Clinical assessment of effects of botanical supplementation on cytochrome P450 phenotypes in the elderly: St John’s wort, garlic oil, Panax ginseng and Ginkgo biloba. Drugs Aging 2005;22:525–539.

102. He X, Luan F, Yang Y, Wang Z, Zhao Z, Fang J, Wang M, Zuo M, Li Y: Passiﬂora edulis: An insight into current researches on phytochemistry and pharmacology. Front Pharmacol 2020;11: 617.

103. National Library of Medicine: Drugs and Lactation Database (LactMed) [Internet]. National Library of Medicine (US), Bethesda, MD, 2006.

104. Zhang H, Li C, Kwok ST, Zhang QW, Chan SW: A review of the pharmacological effects of the dried root of Polygonum cuspidatum (Hu Zhang) and its constituents. Evid Based Complement Alternat Med 2013;2013:208349.

105. Khan M, Maryam A, Mehmood T, Zhang Y, Ma T: Enhancing activity of anticancer drugs in multidrug resistant tumors by modulating P-glycoprotein through dietary nutraceuticals. Asian

Pac J Cancer Prev 2015;16:6831–6839.

(106) Zhang H, Li C, Kwok ST, Zhang QW, Chan SW. A Review of the Pharmacological Effects of the Dried Root of Polygonum cuspidatum (Hu Zhang) and Its Constituents. Evid Based Complement Alternat Med. 2013;2013:208349. doi: 10.1155/2013/208349. Epub 2013 Sep 30. PMID: 24194779; PMCID: PMC3806114.

(107) Khan M, Maryam A, Mehmood T, Zhang Y, Ma T. Enhancing Activity of Anticancer Drugs in Multidrug

Resistant Tumors by Modulating P-Glycoprotein through Dietary Nutraceuticals. Asian Pac J Cancer Prev.

2015;16(16):6831-9. doi: 10.7314/apjcp.2015.16.16.6831. PMID: 26514453.

(108) Rodrigues M, Alves G, Francisco J, Fortuna A, Falcãoa A. Herb-drug Pharmacokinetic Interaction

between Carica Papaya Extract and Amiodarone in Rats J Pharm Pharm Sci (www.cspsCanada.org) 17(3) 302 - 315, 2014

(109) Misaka S, Kawabe K, Onoue S, Werba JP, Giroli M, Tamaki S, Kan T, Kimura J, Watanabe H, Yamada S.

Effects of green tea catechins on cytochrome P450 2B6, 2C8, 2C19, 2D6 and 3A activities in human liver and

intestinal microsomes. Drug Metab Pharmacokinet. 2013;28(3):244-9. doi: 10.2133/dmpk.dmpk-12-rg-101.

Epub 2012 Dec 25. PMID: 23268924.

(110) Misaka S, Kawabe K, Onoue S, Werba JP, Giroli M, Watanabe H, Yamada S. Green tea extract affects

the cytochrome P450 3A activity and pharmacokinetics of simvastatin in rats. Drug Metab Pharmacokinet.

2013;28(6):514-8. doi: 10.2133/dmpk.dmpk-13-nt-006. Epub 2013 May 21. PMID: 23698259.

(111) Fukuda I, Tsutsui M, Sakane I, Ashida H. Suppression of cytochrome P450 1A1 expression induced by

2,3,7,8-tetrachlorodibenzo-p-dioxin in mouse hepatoma hepa-1c1c7 cells treated with serum of (-)-

epigallocatechin-3-gallate- and green tea extract-administered rats. Biosci Biotechnol Biochem. 2009

May;73(5):1206-8. doi: 10.1271/bbb.80868. Epub 2009 May 7. PMID: 19420696.

(112) Engdal S, Nilsen OG. In vitro inhibition of CYP3A4 by herbal remedies frequently used by cancer

patients. Phytother Res. 2009 Jul;23(7):906-12. doi: 10.1002/ptr.2750. PMID: 19170155.

(113) Mooiman KD, Maas-Bakker RF, Hendrikx JJ, Bank PC, Rosing H, Beijnen JH, Schellens JH, Meijerman I.

The effect of complementary and alternative medicines on CYP3A4-mediated metabolism of three different

substrates: 7-benzyloxy-4-trifluoromethyl-coumarin, midazolam and docetaxel. J Pharm Pharmacol. 2014

Jun;66(6):865-74. doi: 10.1111/jphp.12208. Epub 2014 Jan 7. PMID: 24392691.

(114) Netsch MI, Gutmann H, Schmidlin CB, Aydogan C, Drewe J. Induction of CYP1A by green tea extract

in human intestinal cell lines. Planta Med. 2006 May;72(6):514-20. doi: 10.1055/s-2006-931537. PMID:

16773535.

(115) Satoh T, Fujisawa H, Nakamura A, Takahashi N, Watanabe K. Inhibitory Effects of Eight Green Tea

Catechins on Cytochrome P450 1A2, 2C9, 2D6, and 3A4 Activities. J Pharm Pharm Sci. 2016 Apr-Jun;19(2):188-97. doi: 10.18433/J3MS5C. PMID: 27518169.

(116) Williams SN, Pickwell GV, Quattrochi LC. A combination of tea (Camellia senensis) catechins is

required for optimal inhibition of induced CYP1A expression by green tea extract. J Agric Food Chem. 2003

Oct 22;51(22):6627-34. doi: 10.1021/jf030181z. PMID: 14558788.

(117) Fujita T, Kawase A, Niwa T, Tomohiro N, Masuda M, Matsuda H, Iwaki M. Comparative evaluation of

12 immature citrus fruit extracts for the inhibition of cytochrome P450 isoform activities. Biol Pharm Bull.

2008 May;31(5):925-30. doi: 10.1248/bpb.31.925. PMID: 18451520.

(118) Holmberg MT, Tornio A, Hyvärinen H, Neuvonen M, Neuvonen PJ, Backman JT, Niemi M. Effect of

grapefruit juice on the bioactivation of prasugrel. Br J Clin Pharmacol. 2015 Jul;80(1):139-45. doi:

10.1111/bcp.12581. Epub 2015 Jun 1. PMID: 25557052; PMCID: PMC4500333.

(119) Girennavar B, Jayaprakasha GK, Patil BS. Potent inhibition of human cytochrome P450 3A4, 2D6, and

2C9 isoenzymes by grapefruit juice and its furocoumarins. J Food Sci. 2007 Oct;72(8):C417-21. doi:

10.1111/j.1750-3841.2007.00483.x. PMID: 17995595.

(120) Gubbins PO, McConnell SA, Gurley BJ, Fincher TK, Franks AM, Williams DK, Penzak SR, Saccente M.

Influence of grapefruit juice on the systemic availability of itraconazole oral solution in healthy adult

volunteers. Pharmacotherapy. 2004 Apr;24(4):460-7. doi: 10.1592/phco.24.5.460.33350. PMID: 15098799.

(121) Santes-Palacios R, Romo-Mancillas A, Camacho-Carranza R, Espinosa-Aguirre JJ. Inhibition of human

and rat CYP1A1 enzyme by grapefruit juice compounds. Toxicol Lett. 2016 Sep 6;258:267-275. doi:

10.1016/j.toxlet.2016.07.023. Epub 2016 Jul 18. PMID: 27444380.

(122) Schwarz UI, Johnston PE, Bailey DG, Kim RB, Mayo G, Milstone A. Impact of citrus soft drinks relative

to grapefruit juice on ciclosporin disposition. Br J Clin Pharmacol. 2006 Oct;62(4):485-91. doi: 10.1111/j.1365-2125.2005.02519.x. PMID: 16995870; PMCID: PMC1885147.

(123) Grenier J, Fradette C, Morelli G, Merritt GJ, Vranderick M, Ducharme MP. Pomelo juice, but not

cranberry juice, affects the pharmacokinetics of cyclosporine in humans. Clin Pharmacol Ther. 2006

Mar;79(3):255-62. doi: 10.1016/j.clpt.2005.11.010. Epub 2006 Feb 7. PMID: 16513449.

(124) Anlamlert W, Sermsappasuk P, Yokubol D, Jones S. Pomelo enhances cyclosporine bioavailability in

healthy male Thai volunteers. J Clin Pharmacol. 2015 Apr;55(4):377-83. doi: 10.1002/jcph.430. Epub 2014

Dec 30. PMID: 25408261.

(125) Zhang Y , Miao L, Lin L , Ren CY , Liu JX , Cui YM . Repeated administration of Sailuotong, a fixed

combination of Panax ginseng, Ginkgo biloba, and Crocus sativus extracts for vascular dementia, alters

CYP450 activities in rats. 2018 Jan 1;38:125-134. doi: 10.1016/j.phymed.2017.02.007. Epub 2017 Feb 27.

(126) Zadoyan G, Fuhr U. Phenotyping studies to assess the effects of phytopharmaceuticals on in vivo

activity of main human cytochrome p450 enzymes. Planta Med. 2012 Sep;78(13):1428-57. doi: 10.1055/s-

0031-1298536. Epub 2012 May 15. PMID: 22588833.

(127) Wang Y, Ye X, Ma Z, Liang Q, Lu B, Tan H, Xiao C, Zhang B, Gao Y. Induction of cytochrome P450 1A1

expression by ginsenoside Rg1 and Rb1 in HepG2 cells. Eur J Pharmacol. 2008 Dec 28;601(1-3):73-8. doi:

10.1016/j.ejphar.2008.10.057. Epub 2008 Nov 11. PMID: 19022240."

(128) Sparreboom A, Cox MC, Acharya MR, Figg WD. Herbal remedies in the United States: potential

adverse interactions with anticancer agents. J Clin Oncol. 2004 Jun 15;22(12):2489-503. doi:

10.1200/JCO.2004.08.182. PMID: 15197212. doi: 10.1200/JCO.2004.08.182.

(129) Klotz S, U. Drug Interactions with Herbal Medicines. Clin Pharmacokinet 2012; 51 (2): 77-104 0312-

5963/12/0002-0077

(130) Malati C, Robertson SM, Hunt JD, Chairez C, Alfaro RM, Kovacs JA, Penzak SR. Pharm.D.Influence of

Panax ginseng on Cytochrome P450 (CYP)3A and Pglycoprotein (Pgp) Activity in Healthy Subjects. J Clin

Pharmacol. 2012 June ; 52(6): . doi:10.1177/0091270011407194

(131) Bogacz A, Mrozikiewicz PM, Karasiewicz M, Bartkowiak-Wieczorek J, Majchrzycki M, Mikolajczak PL,

Ozarowski M, Grzeskowiak E. The influence of standardized Valeriana officinalis extract on the CYP3A1 gene

expression by nuclear receptors in in vivo model. Biomed Res Int. 2014;2014:819093. doi:

10.1155/2014/819093. Epub 2014 Sep 11. PMID: 25302309; PMCID: PMC4180645.

(132) Hellum BH, Hu Z, Nilsen OG. Trade herbal products and induction of CYP2C19 and CYP2E1 in cultured

human hepatocytes. Basic Clin Pharmacol Toxicol. 2009 Jul;105(1):58-63. doi: 10.1111/j.1742-

7843.2009.00412.x. Epub 2009 Apr 3. PMID: 19371257.

(133) Strandell J, Neil A, Carlin G. An approach to the in vitro evaluation of potential for cytochrome P450

enzyme inhibition from herbals and other natural remedies. Phytomedicine. 2004 Feb;11(2-3):98-104. doi:

10.1078/0944-7113-00379. PMID: 15070158.
